# Diaroyl Tellurides: Synthesis, Structure and NBO Analysis of (2-MeOC_6_H_4_CO)_2_Te – Comparison with Its Sulfur and Selenium Isologues. The First Observation of [MgBr][R(C=Te)O] Salts

**DOI:** 10.3390/molecules14072555

**Published:** 2009-07-13

**Authors:** Osamu Niyomura, Shoho Nakaiida, Ryo Yamada, Shinzi Kato, Masaru Ishida, Masahiro Ebihara, Fumio Ando, Jugo Koketsu

**Affiliations:** 1 Department of Chemistry, School of Engineering, Gifu University, Gifu, Japan; 2 Department of Applied Chemistry, College of Engineering, Chubu University, Kasugai, Japan

**Keywords:** diacyl telluride, diacyl selenide, diacyl sulfides, Grignard reagent, magnesium carbotelluroate

## Abstract

A series of aromatic diacyl tellurides were prepared in moderate to good yields by the reactions of sodium or potassium arenecarbotelluroates with acyl chlorides in acetonitrile. X-ray structure analyses and theoretical calculations of 2-methoxybenzoic anhydride and bis(2-methoxybenzoyl) sulfide, selenide and telluride were carried out. The two 2-MeOC_6_H_4_CO moieties of bis(2-methoxybenzoyl) telluride are nearly planar and the two methoxy oxygen atoms intramolecularly coordinate to the central tellurium atom from both side of C(11)-Te(11)-C(22) plane. In contrast, the oxygen and sulfur isologues (2-MeOC_6_H_4_CO)_2_E (E = O, S), show that one of the two methoxy oxygen atoms contacts with the oxygen atom of the carbonyl group connected to the same benzene ring. The structure of di(2-methoxybenzoyl) selenide which was obtained by MO calculation resembles that of tellurium isologues rather than the corresponding oxygen and sulfur isologues. The reactions of di(aroyl) tellurides with Grignard reagents lead to the formation of tellurocarboxylato magnesium complexes [MgBr][R(C=Te)O].

## Introduction

In contrast to diacyl sulfides and selenides, the isolation of diacyl tellurides is very difficult due to their instability toward oxygen and thermal conditions. The first isolation of diacyl telluride was reported in 1978 by Bergman and Engman, who isolated phthaloyl telluride [1]. In 1985, du Mont and his coworkers successfully isolated two aliphatic diacyl tellurides [(RCO)_2_Te: R = Me, *t*-Bu] by using (Me_3_Si)_2_Te as a tellurium source [2] and reported a molecular structural analysis of di(adamanthoyl) tellurides [3]. In 1986 we also reported the isolation of two aromatic diacyl tellurides from the reaction of sodium carbotelluroate with acyl chlorides [4]. In this paper, we report an improved synthesis and some reactions of diaroyl tellurides, and NBO (natural bond orbital) analysis of (2-MeOC_6_H_4_)_2_E (E= O, S, Se, Te). 

## Results and Discussion

### Synthesis

The reactions of sodium telluride with two molar equivalents of acyl chlorides in ether, benzene and tetrahydrofuran etc. gave di(acyl) tellurides **4** in yields below 50% [5], due to the difficulty of separating by-products such as diacyl ditelluride, acid anhydride and black tellurium. We then found that the use of acetonitrile as a solvent led to good yields of **4**. For example, two molar equivalents of 4-methylbenzoyl chloride in acetonitrile were added dropwise at 0 ºC to a suspension of freshly prepared sodium telluride in the same solvent, and the mixture was stirred for 2 h. The precipitates and excess of sodium telluride were filtered out and the solvent was removed under reduced pressure to give bis(4-methylbenzoyl) telluride (**4e**) in 54% yield. 

**Scheme 1:**
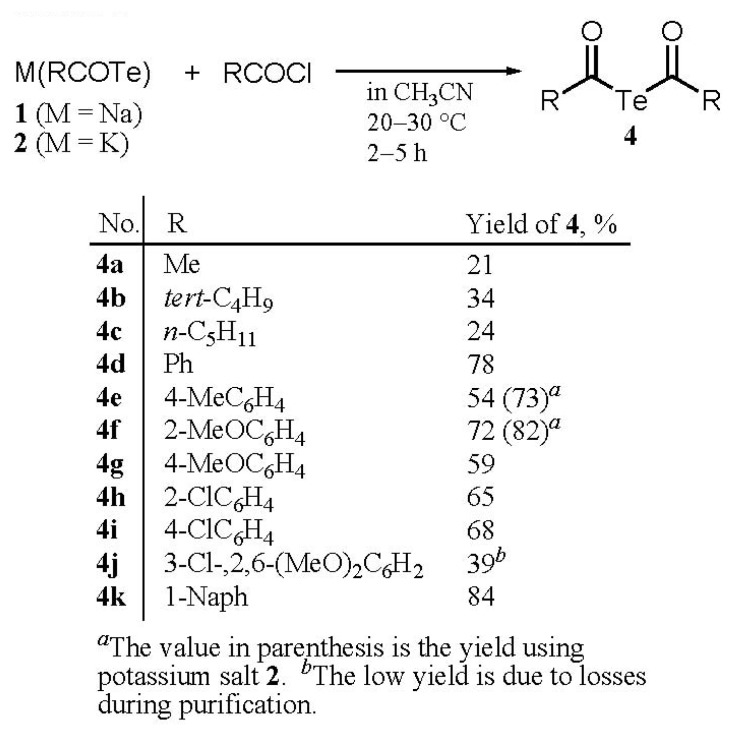
Synthesis of diacyl tellurides **4a-k**.

Under similar conditions, the reactions of other sodium or potassium arenecarbotelluroates with the corresponding aroyl chlorides led to the expected diacyl tellurides **4d, 4f‒4k** in isolated yields of 39‒84% (Scheme 1). In contrast, the preparation of aliphatic compounds **4a‒4c** resulted in low yields of 21‒34%, most likely due to their instability.

Compounds **4** are pale yellow to yellow liquids or crystals that readily dissolve in common aprotic solvents such dichloromethane and chloroform. They are susceptible to degradation by oxygen, although the aromatic compounds appear to be much more stable in this respect than the aliphatic ones. For example, while 4-methylbenzoyl derivative **4e** can be stored without an appreciable change for 3 hours under exposure to air at 15 °C, and it is stable for at least a week under oxygen-free conditions at 0 °C. Upon exposure to air under similar conditions, aliphatic diacyl tellurides **4a-4c** quickly decomposed to give the corresponding acid anhydrides with the liberation of black tellurium.

### Molecular structures of bis(2-methoxybenzoyl) chalcogenides

**Figure 1:**
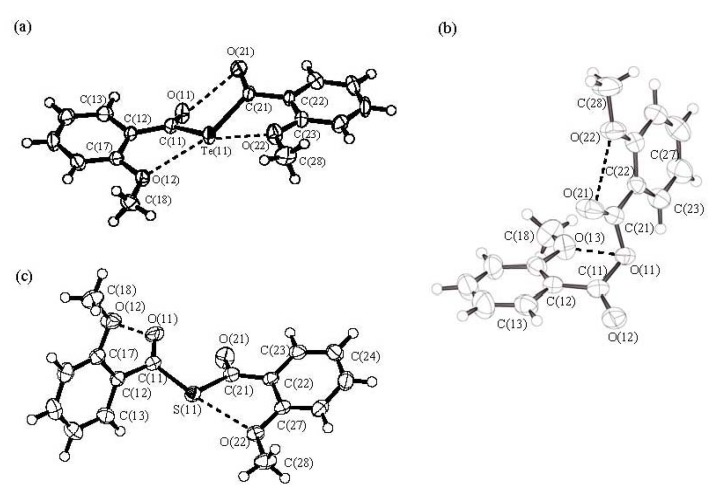
ORTEP drawings of (a) bis(2-methoxybenzoyl) telluride (**4f**), (b) 2-methoxy-benzoic anhydride (**5**) and (c) bis(2-methoxybenzoyl) sulfide (**6**). The thermal ellipsoid plots represent 50% probability.

Figure 1 shows the ORTEP drawing of (2-MeOC_6_H_4_)_2_Te (**4f**) along with those of the corresponding oxygen and sulfur isologues (2-MeOC_6_H_4_CO)_2_O (**5**) and (2-MeOC_6_H_4_CO)_2_S (6). The final atomic positional parameters are shown in Table 1. Selected bond distances and angles are listed in Table 2.

**Table 1:** Crystal date and data collection of bis(2-methoxybenzoyl) telluride (**4f**) [8], 2-methoxybenzoic anhydride (**5**) [8], and bis(2-methoxybenzoyl) sulfide (**6**) [8].

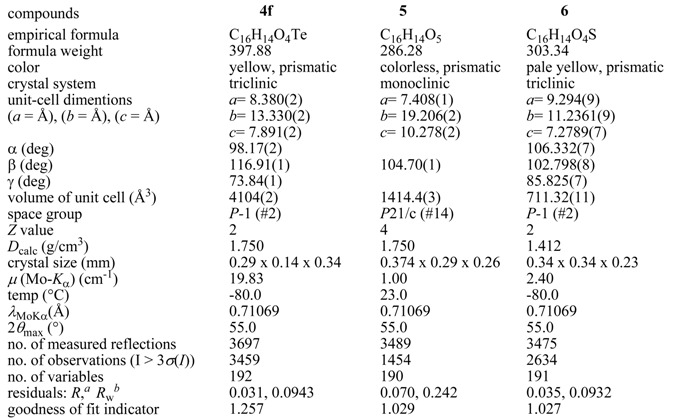

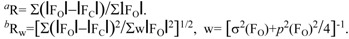

**Table 2:** Selected bond distances (A), angles (^o^) and torsion angles (^o^) of bis(2-methoxy-benzoyl) telluride (**4f**), 2-methoxybenzoic anhyride (**5**) and bis(2-methoxybenzoyl) sulfide (**6**).

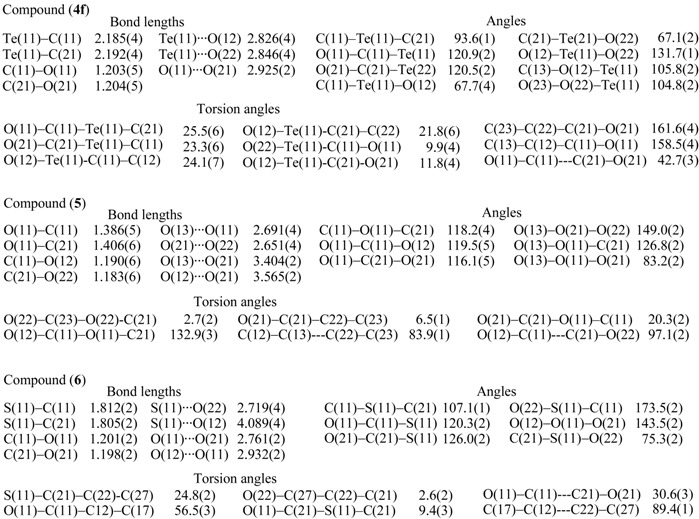

The two 2-MeOC_6_H_4_CO moieties of **4f** are nearly planar [torsion angles: C(21)-C(22)-C(23)-O(22) = –5.3°; C(11)-C(12)-C(13)-O(12) = –5.1°], with the two carbonyl groups in the same direction and connected to the central tellurium atom. The interplanar angle involving two carbonyl groups O(11)C(11)---C(21)O(21) is 42.6°, which is significantly larger than that of di(1-adamantoyl) telluride (9.6°) [3]. The distance (2.925 Å) between the two carbonyl oxygen atoms [O(11)---O(21)] is somewhat shorter than the sum of the van der Waals radii of both atoms (3.04 Å) [6], which suggests weak intramolecular interaction. The two methoxy groups occupy the position opposite the carbonyl groups. The distances between the methoxy oxygen and central tellurium atoms [O(22)---Te(11) and O(21)---Te(11) (2.826 Å and 2.846 Å, respectively) are significantly shorter than the sum of the van der Waals radii of both atoms (3.58 Å) [6], which suggests intramolecular nonbonding orbital interactions [7].

For comparison, we carried out an X-ray structure analysis of 2-methoxybenzoic anhydride and thioanhydride, **5** and **6**. One benzene ring [C(12)–C(13)] and the carboxyl group [O(11), C(21), O(21)] in 5 or the thiocarboxyl group [S(11), C(21), O(21)] in 6 are not coplanar. In both compounds **5** and **6**, the distances between one of the two methoxy oxygen atoms and one of the two carbonyl oxygen atoms are short: 2.651 Å [O(21)---O(22) ] in **5** and 2.932 Å [O(11)---O(12) ] in **6**, respectively. The distances between the central oxygen and the methoxy oxygen atoms [O(11)---O(13)] in **5** and between the central sulfur and the methoxy oxygen [S(11)---O(22)] in **6** are 2.691 Å and 2.719 Å, respectively. These values are shorter than the sum (3.04 Å) [6] of the van der Waals radii of both atoms, which suggests O---O intramolecular nonbonding interaction. On the other hand, the two carbonyl groups in thioanhydride **6** are in the same direction, with an interplanar angle [O(11)–C(11)–C(21)–O(21) of 30.5(1)° and the distance between the two carbonyl oxygen atoms [O(11)---O(21)] is only 2.761(2) Å, which suggests an O---O intramolecular nonbonding interaction. The alignment of O(22)–S(11)–C(11) is nearly linear: 173.4 (7)° [7,9].

**Figure 2:**
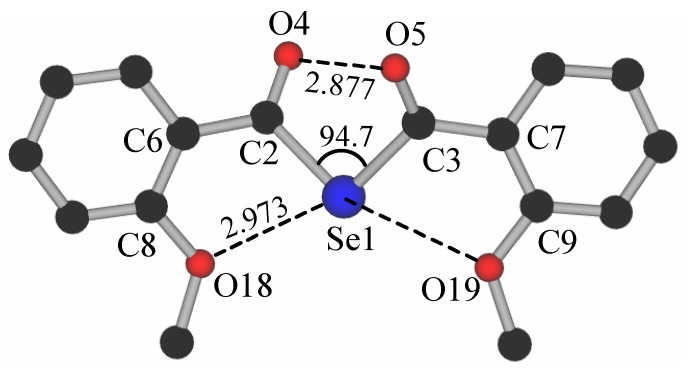
The optimized structure of (2-MeOC_6_H_4_CO)_2_Te (**7**) calculated at the RHF/LANL-2dz level. Distances are in angstrom (Å) and angles (°). Torsion angles: O4-C2-C3-O5 = 63.5°; C2-Se1-O18-C8 = 24.2° in degrees (for the other distances and angles: see Table 3).

We were unable to obtain single-crystals of the selenium isologue **7** even after several attempts. Therefore, the structure of **7** was optimized by using the structure parameters of bis(2-methoxybenzoyl) diselenide [10]. As shown in Figure 2, the structure of **7** resembles that of tellurium isologue **4f** rather than the corresponding sulfur isologue **6**, although the 2-MeOC_6_H_4_ and C=O groups in the 2-MeOC_6_H_4_CO moiety are not planar [torsion angles: O(4)–C(2)–C(6)–C(8) = 29.5°]. Thus, the two carbonyl groups are in the same direction with torsion angles [O(4)-C(2)---C(3)-O(5)] of 63.1°. The distance between the two carbonyl oxygen atoms [O(4)---O(5)] (2.877 Å) is somewhat shorter than the sum of the van der Waals radii of the oxygen atoms (3.04 Å) [6]. The two methoxy oxygen atoms contact to the central selenium atom and both distances, O(18)---Se(1) and O(19)---Se(1), are short [2.973 Å], indicating intramolecular nonbonding interactions, as in **4f**.

The C-E-C and O=C---C=O interplanar angles in the -CO-E-CO- moieties of (2-MeOC_6_H_4_CO)_2_E (E = O, S, Se, Te) are shown in Table 3. Both the C-E-C angles and O=C---C=O interplanar angles are narrow, in the order E = O > S > Se > Te. Narrowing of the C-Te-C angle reduces the intramolecular repulsion of the carbonyl oxygen atoms.

**Table 3:** C=O---O=C distances and < C-E-C and interplanar angles (*ϕ*O=C---C=O) in (2- MeOC_6_H_4_CO)_2_E (E= O, S, Se, Te).

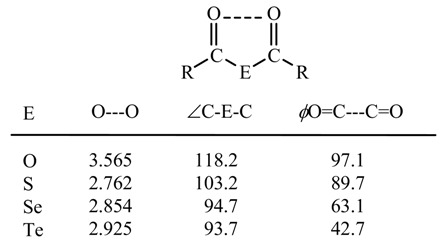

### Packing

**Figure 3:**
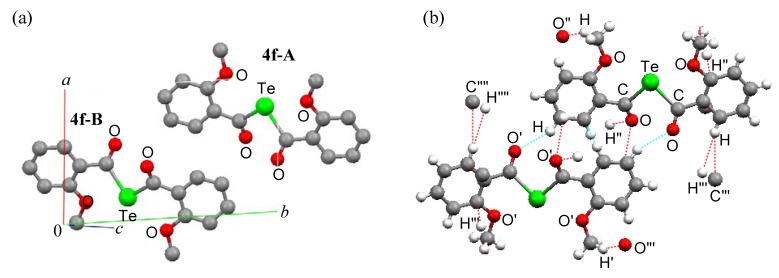
(a) Packing and (b) intermolecular short contacts of (2-MeOC_6_H_4_CO)_2_Te (**4f**), view down to *c*-axis. Green, red, yellow and gray balls are tellurium, oxygen, sulfur and carbon atoms, respectively. For (a), hydrogen atoms are omitted for clarity and molecules **4f-A** and **4f-B** are enantiomorphic with each other. For (b), red and light blue dotted lines show intermolecular short contacts.

**Figure 4:**
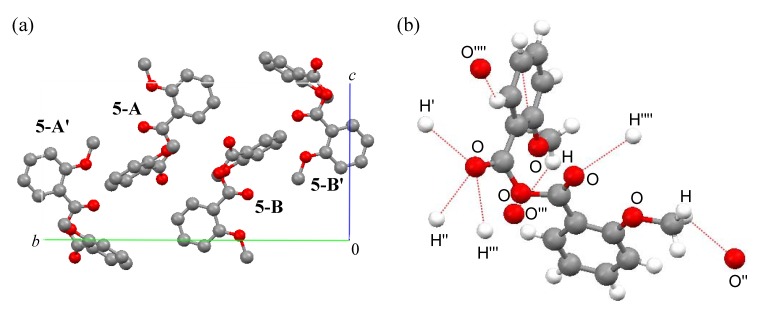
(a) Packing and (b) intermolecular short contacts of (2-MeOC_6_H_4_CO)_2_O (5) view down to *a*-axis. Red, gray and white balls are oxygen, carbon and hydrogen atoms, respectively. For (a), hydrogen atoms are omitted for clarity and molecules 5-A, **5-A'**, **5-B** and **5-B'** are enantiomorphic with each other. For (b), red dotted lines show intermolecular short contacts.

**Figure 5:**
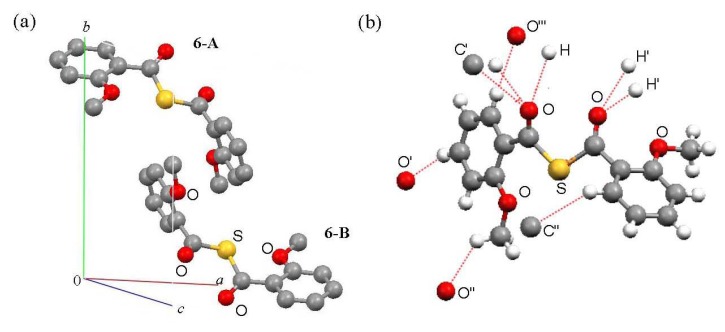
(a) Packings and (b) intermolecular short contacts of (2-MeOC_6_H_4_CO)_2_S (**6**) view down to *a*-axis. Yellow, red, gray and white balls are sulfur, oxygen, carbon and hydrogen atoms, respectively. For (a), hydrogen atoms are omitted for clarity and molecules **6-A** and **6-B** are enantiomorphic with each other. For (b), red dotted lines show intermolecular short contacts.

The unit cells and intermolecular short contacts of compounds **4f**, **5** and **6** are shown in Figure 3, Figure 4 and Figure 5, respectively. Compounds **4f** and **6** exist as two molecules in a unit cell, while compound 5 exists as four molecules [Figure 3, (a); Figure 4, (a); Figure 5, (a)]. Structures **4f-A** and **4f-B**, **5-A**, **5-A’**, **5-B** and **5-B’**, and **6-A** and **6-B** are enantiomorphic. Their 3-D packing structures are formed by weak hydrogen-bond interactions between the carbonyl oxygen and the aromatic ring or methoxy methyl protons and CH-(π) interactions between the methoxy methyl hydrogen and benzene-ring carbon atoms [Figure 3, (b); Figure 4, (b); Figure 5, (b)] [11].

### Ab initio calculations

To elucidate the nature of the nonbonding attractions, *ab initio* geometry optimizations were performed at the B3LYP/6-311G(2d,p) level for compounds **5** and sulfur **6** and at the RHF/LANL2DZ level+p for compounds **4f** and **7** with the Gaussian 03 program [12,13] using their X-ray geometries [10,14]. The Beck-style three-parameter density functional theory [15] with the Lee/Yang/Parr [16] gradient-corrected correlation function was used in our calculation. Effective core potentials (ECP) with an appropriate valence basis set [LANL2DZ+polarization functions (d) and diffusion functions (sp)] were used for Te, Se, S and O, and the 04-31G* basis set was used for C and H [17]. At all levels and with all of the basis sets used, two conformational energy minima were seen for (2-MeOC_6_H_4_CO)_2_E [E = O (5), S (6), Se (7), Te (**4f**)], which are conformers with RCO groups [*syn*- (*C*_s_ symmetry) and *anti*-conformer (*C*_2_ symmetry)] (Figure 5). The structure parameters of the optimized geometries of these compounds are consistent with the absence of intermolecular interactions in crystal structures with these XRD values (detail data: *ESI-Table 1*).

To understand the orbital interactions of the -C(O)EC(O)- (E = O, S, Se, Te) moiety, the NBO analysis of (2-MeOC_6_H_4_CO)_2_E [E = O (**5**), S (**6**), Se (7), Te (**4f**)] was performed using the X-ray geometries of **5** and **6** (Table 4). For the -C(O)EC(O)- moiety, orbital interactions are observed for the lone-pair electrons, which indicates delocalization of the lone-pair electrons on the carbonyl oxygen to the π* of a chalcogen atom (E) – carbon bond and on the central atom (E) to the π* orbital of the carbonyl groups. In addition, for 4f, there are nonbonding intramolecular interactions *n*_O2_→σ*_Te-C2_ and *n*_O4_→σ*_Te-C1_, which indicates a large stabilization compared to other derivatives without an ortho-methoxy substituent.

**Table 4:** NBO analysis of (2-MeOC_6_H_4_CO)_2_E [E = O (**5**), S (**6**), Se (**7**), Te (**4f**)].

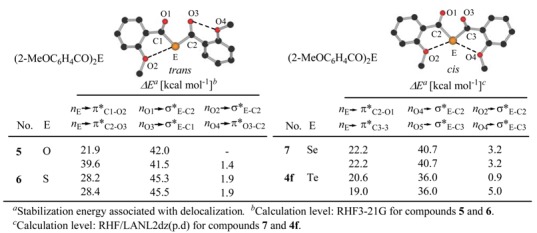

### Reactions diacyl tellurides with Grignard reagents

**Scheme 2:**
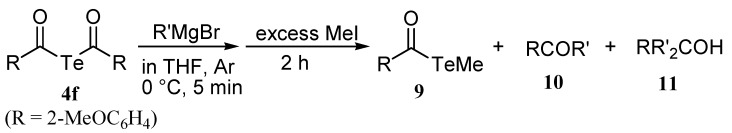
Reactions of bis(2-methoxybenzoyl) telluride (**4f**) with Grignard reagents.

Compound **4f** readily reacts with sodium methoxide and piperidine at 0 °C to yield the corresponding sodium (**1**, R = 2-MeOC_6_H_4_) and piperidinium salts (**8**) [C_5_H_10_N^+^ (2-MeOC_6_H_4_COTe)^-^], respectively [4b]. It is well known that the treatment of aromatic acid anhydrides such as benzoic anhydride with RMgBr (R = aryl), followed by hydrolysis, gives the corresponding carboxylic acid and triarylmethanol [18]. Previously, we had observed that bis(4-methylbenzoyl) sulfide and selenide readily react with 4-methylphenylmagnesium bromide, and subsequent HCl-acidolysis gives tris(4-methylphenyl)carbinol, along with the corresponding thio- [19] and selenocarboxylic acid salts [20a, 20b]. As expected, bis(2-methoxybenzoyl) telluride (**4f**) reacted with arylmagnesium bromides at 0 ºC to afford the corresponding ketone and triarylmethanol **11** in moderate yields (Scheme 2, Table 5). However, the reaction with *t-*butylmagnesium bromide under similar conditions gave bis(2-methoxybenzoyl) ditelluride (**12**) [10], along with 2-methoxybenzoic acid.

Interestingly, when a tetrahydrofuran solution of phenylmagnesium bromide was added, the orange-colored solution of **4f** immediately changed to dark green. The electron and ^13^C-NMR spectra of this dark green solution showed a characteristic absorption maximum at λ_max_ 732 nm and a new signal at δ 232 ppm, respectively. In addition, treatment of the dark green solution with excess of iodomethane led to *Te*-methyl 2-methoxybenzenecarbotelluroate (**9**). Several attempts to isolate the dark green compound with an absorption maximum at λ_max_ 732 nm were unsuccessful. As for the cause of this change, du Mont and his coworkers have reported that the *n*-λ* transitions of the C=Te group of *O*-trimethylsilyl ethanecarbotelluroate appear at 732 nm [2a]. We have also found that *O*-triorganylsilyl arenecarbotelluroates [20a] and aromatic carbotelluroic OH-acid (ArCTeOH) in polar solvents [20b] show a similar dark green to deep blue coloration and that their *n*-π* transitions of the C=Te group and tellurocarbonyl carbon chemical shifts appear in the ranges of 600‒750 nm and δ 230‒250 ppm, respectively [20]. 

**Table 5:** Reactions of (2-MeOC_6_H_4_CO)_2_ Te (**4f**) with Grignard reagents.

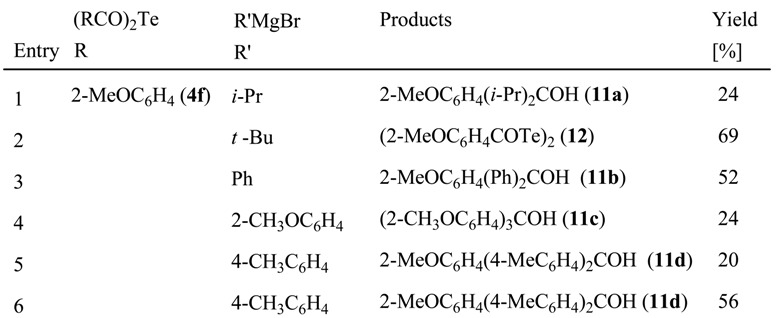

**Scheme 3:**
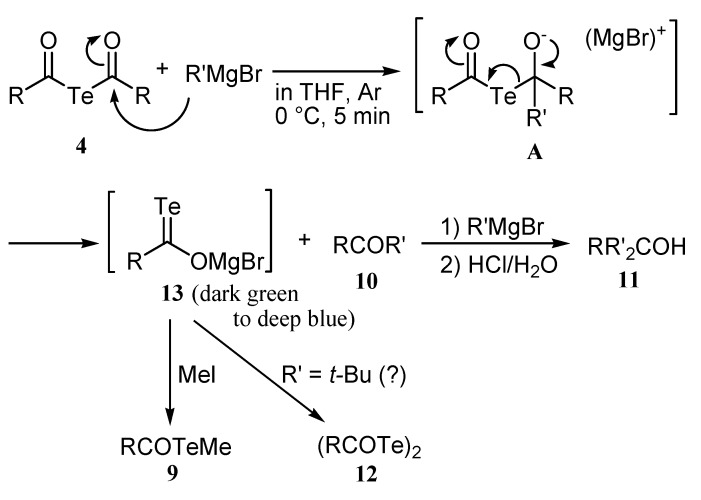
A possible mechanism for the reactions of (2-MeOC_6_H_4_CO)_2_ Te (**4f**) with Grignard reagents.

Presumably, the absorption maximum at 732 nm observed in the reaction of bis(2-methoxybenzoyl) telluride with Grignard reagent may be attributable to the *n*-π* transitions of the C=Te group, indicating the formation of a complex **13**, in which the oxygen atom of the carbotelluroato moiety is connected to the central magnesium ion. (Scheme 3) At present stage, it is unclear whether or not the ditelluride **12** (R = 2-MeOC_6_H_4_) forms *via* **13**, since no color change to dark green or deep sky blue was observed when *t*-BuMgBr was added.

## Conclusions

The use of acetonitrile as a solvent was found to be effective for the synthesis of aromatic diacyl tellurides from the reaction of acyl chlorides with sodium and potassium tellurides. X-ray crystal structure analyses and theoretical calculations for 2-methoxybenzoic anhydride (**5**) and bis(2-methoxybenzoyl) sulfide (**6**), selenide **7** and telluride **4f** revealed that the two 2-MeOC_6_H_4_CO moieties of **4f** are nearly planar, where the two carbonyl oxygen atoms are in the same direction, and the two methoxy oxygen atoms intramolecularly coordinate to the central tellurium atom from both sides of the C(11)–Te(11)–C(22) plane. For the corresponding acid anhydride **5** and diacyl sulfide **6**, one of the two methoxy oxygen atoms is in contact with the central oxygen or sulfur atom and the other methoxy oxygen atom is in contact the oxygen atom of a carbonyl group connected to the same benzene ring. The structure of bis(2-methoxybenzoyl) selenide (**7**) obtained by theoretical calculations resembles that of tellurium isologue **4f**, rather than the corresponding oxygen and sulfur isologues **5** or **6**. Thus, the two carbonyl oxygen atoms are in the same direction and the two methoxy oxygen atoms intramolecularly contact the central selenium atom from both sides of the C(11)–Se(11)–C(22) plane. Natural bond orbital (NBO) analysis of the telluride **4f** and selenide **7** revealed that two types of orbital interactions, *n*_O2_→σ*_E-C2_/*n*_O4_→σ*_E-C1_ and *n*_O2_→σ*_E-C1_/*n*_O4_→σ*_E-C2_, are important and the former particularly play a predominant role. The reactions of diaroyl tellurides with Grignard reagents led to the formation of the corresponding carbotelluroato magnesium complexes in which the oxygen atoms of carbotelluroato ligands are connected to the magnesium ion. 

## Experimental

### General

The melting points were measured by a Yanagimoto micromelting point apparatus and uncorrected. The IR spectra were measured on a PERKIN ELMER FT-IR 1640 and a JASCO grating IR spectrophotometer IR-G. The 1H-NMR spectra were recorded on JEOL JNM-GX 270 (270 MHz) or JNM-α400 (399.7 MHz) instruments, respectively, with tetramethylsilane as an internal standard. The 13C-NMR spectra were obtained from a JEOL JMN-GX 270 (67.9 MHz). The 125Te NMR spectra for aromatic diacyl tellurides **4d‒4k** were obtained from a JEOL JNM-GX-270 (85.9 MHz) with Ph_2_Te as an external standard: their chemical shifts were determined relative to Me_2_Te with δ (Ph_2_Te) = 420 ppm relative to Me_2_Te. The 125Te-NMR spectra for aliphatic diacyl tellurides **4a-4c** were obtained on a JEOL JNM-α400 (126.0 MHz) instrument with dimethyl telluride as an external standard. The electron spectra were obtained with Hitachi 124 and 310 spectrometers. The mass spectra were recorded on Shimadzu GCMS QP1000 (A) (EI/CI, model) mass spectrometers. The high resolution mass spectra (HRMS) were recorded on Shimadzu GCMS 9020DF high resolution mass spectrometer.

### Materials

Sodium [20c] and potassium carbotelluroates **1** and **2** [4b,20d] were prepared according to the literature. Diethyl ether and hexane were refluxed and distilled from sodium metal using benzophenone as indicator before use. Dichloromethane and acetonitrile were distilled over phosphorus pentoxide. All manipulations were carried out under argon.

### X-ray Measurements [20,21,22, 23 and 24]

The measurements were carried out on a Rigaku AFC7R four-circle diffract meter with graphite-monochromated Mo-K radiation (= 0.71069 Å). All of the structures were solved and refined using the teXsan crystallographic software package on an IRIS Indigo computer. X-ray quality crystals of **4f**, **5** and **6** were obtained by recrystallization from ether/petroleum ether. The crystal of **4f** was cut from the grown needles and coated with an epoxy resin and mounted on a glass fiber. The cell dimensions were determined from a least-squares refinement of the setting diffract-meter angles for 25 automatically centered reflections. The crystals of **5** and **6** were obtained by recrystallization from a mixed solvent of ether/hexane. Lorentz and polarization corrections were applied to the data, and empirical absorption corrections (*ψ*-scans [21]) were also applied. The structures were solved by direct methods using SIR97 [22] and refined by using SHELXL97 [23]. Scattering factors for neutral atoms were from Cromer and Waber [24] and anomalous dispersion [25] was used. The final full-matrix least-squares cycle included nonhydrogen atoms with anisotropic thermal parameters. H atoms were placed in idealized positions and treated as riding atoms with C–H distances in the range 0.93–0.98 Å.

### Preparation of (2-MeOC_6_H_4_CO)_2_S (**5**) [26] and (2-MeOC_6_H_4_CO)_2_O (**6**) [27]

These compounds were synthesized following the indicated literature procedures and recrystallized from benzene/petroleum ether. Compound **5**: M.p. 66–69 °C; IR (KBr) [cm^-1^]: 1,732 [C=O], 1,662 [C=O]; ^1^H-NMR (399.7 MHz, CDCl_3_): δ = 3.21 (s, 3H) [C*H*_3_O], 6.96 (d, *J* = 7.9 Hz, 2H) [arom], 7.00 (t, *J* = 7.9 Hz, 2H) [arom], 7.48 (t, *J* = 7.9 Hz, 2H) [arom], 7.71 (d, *J* = 7.9 Hz, 2H) [arom]; ^13^C-NMR (67.9 MHz, CDCl_3_): δ = 55.8 [*C*H_3_O], 112.0, 120.4, 127.4, 130.2, 134.2, 157.8 [arom], 192.1 [*C*=O]. Compound **6**: M.p. 73-74 °C. IR (KBr) [cm^-1^]: 1,733 [C=O], 1,700 [C=O]; ^1^H-NMR (399.7 MHz, CDCl_3_): δ = 3.85 (s, 6H) [C*H*_3_O], 7.00 (d, *J* = 8.0 Hz, 2H, [arom]), 7.03 (t, *J* = 8.0 Hz, 2H, [arom]), 7.55 (t, *J* = 8.0 Hz, 2H, [arom]), 8.01 (d, *J* = 8.0 Hz, 2H, [arom]); ^13^C-NMR (67.9 MHz, CDCl_3_): δ = 55.9 [*C*H_3_O], 112.1, 118.1, 120.3, 133.0, 135.2 [arom], 161.9 [*C*=O].

### Syntheses of di(acyl) tellurides **4a-k**

The preparation of bis(4-methylbenzoyl) telluride (**4e**) is described in detail as a typical procedure. Except were indicated, potassium carbotelluroates **2** were used for the preparation of compounds **4**. The IR spectra of the latter were consistent with those of the corresponding authentic samples, respectively, which were prepared by the reaction of the corresponding piperidinium or potassium salts with acyl chlorides. 

*Diacetyl telluride* (**4a**) [2a]. Acetyl chloride (2.01 g, 10.3 mmol) was added to a suspension of freshly prepared sodium methanecarbotelluroate (0.808 g, 10.3 mmol) in acetonitrile (15 mL) and the mixture was stirred at 0 °C for 30 min. The resulting precipitates were filtered out. Removal of the solvent under reduced pressure and vacuum distillation of the residue gave 1.426 g (65 %) of crude diacetyl telluride (**4a**) as yellow liquid. Vacuum distillation of the resulting residue gave 0.37 g (21 %) of **4a** as a pale yellow liquid. B.p. 88–90 °C/25 torr; IR (CsI, neat) [cm^-1^]: 3,410, 3,000, 2,900, 1,821, 1,755 [C=O], 1,710 [C=O], 1,410, 1,350, 1,230, 1,070, 550; ^1^H-NMR (270 MHz, CDCl_3_): δ = 2.68 (s, [C*H*_3_]); ^13^C-NMR (67.8 MHz, CDCl_3_): δ = 42.6 [*C*H_3_], 196.6 [*C*=O]; ^125^Te-NMR (85.9 MHz, CDCl_3_): δ = 1035.5; MS (CI, 70 eV), *m/e* = 218 [M^+^ +1], 216, 214; HRMS (EI, 20 eV) calcd. for C_4_H_6_O_2_Te: *m/z =* 215.9430; Found: 215.9447.

*Bis(2,2-dimethylpropanoyl) telluride* (**4b**). Yield: 34%; Pale yellow liquid; b.p. 72–74°C/0.2 torr; IR (CsI, neat) [cm^-1^]: 2,980, 1,745 [C=O], 1,695 [C=O], 1,470, 1,450, 1,390, 1,360, 1,220, 1,025, 1,000, 865, 765, 580; ^1^H-NMR (270 MHz, CDCl_3_): 1.15 (s, 18H) [C*H*_3_]; ^13^C-NMR (67.8 MHz, CDCl_3_): δ = 26.10 (*C*H_3_), 53.7 [*C*CO], 206.1 [*C*=O]; ^125^Te-NMR (85.9 MHz, CDCl_3_): δ = 1013.7; MS (CI, 70 eV): *m/z* = 301 [M^+^ +1], 299, 297, 85 [C_4_H_9_CO]; HRMS (EI, 20 eV) calcd. for C_10_H_18_O_2_Te: *m/z =* 300.03689; Found: 300.03695.

*Dihexanoyl telluride* (**4c**). Yield: 24%; Pale yellow liquid; b.p. 75–78°C/0.15 torr; IR (CsI, neat) [cm^-1^]: 2,950, 2,900, 1,750 [C=O], 1,700 [C=O], 1,470, 1,120 m 1,040, 720; ^1^H-NMR (270 MHz, CDCl3): δ = 0.90 (t, *J* = 7.3 Hz, 3H) [C*H*_3_], 1.33 (m, 4H) [C*H*_2_], 1.67 (m, 2H) [C*H*_2_], 2.85 (t, *J* = 7.3 Hz, 2H) [C*H*_2_]; ^13^C-NMR (67.8 MHz, CDCl_3_): δ = 13.8 [*C*H_3_], 22.4, 24.4, 31.0 [*C*H_2_], 56.0 [*C*H_2_CO], 200.5 [*C*=O]; ^125^Te-NMR (85.9 MHz, CDCl_3_): δ = 1111.7; MS (CI, 70 eV): *m/z* = 329 [M^+^ +1], 327, 325, 99 (C_5_H_11_CO); HRMS (EI, 20 eV) calcd. for C_12_H_22_O_2_Te: *m/z =* 328.06842; Found: 328.06826.

*Dibenzoyl telluride* (**4d**). 78%. Orange yellow microfine crystals (recrystallization solvents: dichloromethane/petroleum ether). M.p. 70–71°C (dec.); IR (KBr) [cm^-1^]: 3,020, 3,010, 1,710 [C=O], 1,675 [C=O], 1,590, 1,575, 1,485, 1,440, 1,300, 1,235, 1,160, 1,070, 1,055, 1,000, 940, 845, 760, 695, 675, 665, 630, 600, 585; ^1^H-NMR (270 MHz, CDCl_3_): δ = 7.438.18 (m, 10 H) [arom]; ^13^C-NMR (67.8 MHz, CDCl_3_): δ = 128.2, 129.0, 134.4, 141.6 [arom], 192.1 [*C*=O]; ^125^Te-NMR (85.9 MHz, CDCl_3_): δ = 1054.7.

*Preparation of bis(4-methylbenzoyl) telluride* (**4e**): From sodium 4-methylbenzenecarbotelluroate*.* 4-Methylbenzoyl chloride (0.357 g, 2.32 mmol) in acetonitrile (5 ml) was added to a suspension of sodium 4-methylbenzenecarbotelluroate (**1**, R = 4-CH_3_C_6_H_4_) (0.680 g, 2.52 mmol) in ether (20 mL) and the mixture was stirred at 0 °C for 2 h. The solvents were evaporated under reduced pressure and ether (50 mL) was added. The precipitates (NaCl and excess of the sodium salt) were filtered out. Removal of the ether under reduced pressure and recrystallization of the residue from dichloromethane/petroleum ether gave 0.497 g (54%) of bis(4-methylbenzoyl) telluride (**4e**) as orange yellow micro crystals. M.p. 83–86 °C (dec.); IR (KBr) [cm^-1^]: 3,020, 2,928, 1,741 [C=O], 1,706 [C=O], 1,690 [C=O], 1,599, 1,548, 1,404, 1,308, 1,198, 1,165, 1,122, 1,039, 1,016, 973, 869, 853, 825, 814, 772, 709, 604, 593, 463; ^1^H-NMR (270 MHz, CDCl_3_): δ = 2.38 (s, 3H) [C*H*_3_], 7.24 (d, *J* = 8.2 Hz, 2H) [arom], 7.69 (d, *J* = 8.2 Hz, 2H) [arom]; ^13^C-NMR (67.8 MHz, CDCl_3_): δ = 21.7 (*C*H_3_), 128.5, 129.6, 139.3, 145.5 [arom], 191.6 [*C*=O]; ^125^Te-NMR (85.9 MHz, CDCl_3_): δ = 1038.9. 

From potassium 4-methylbenznecarbotelluroate*.* Similar to the sodium salt, the reaction of 4-methylbenzoyl chloride (0.300 g, 1.95 mmol) with potassium 4-methylbenzenecarbotelluroate (**2**, R = 4-CH_3_C_6_H_4_) (0.561 g, 1.95 mmol) gave 0.520 g (73%) of **4e**.

*Bis(2-methoxybenzoyl) telluride* (**4f**): From sodium 2-methoxybenzenecarbotelluroate*.* Similarly to the synthesis of **4e**, the reaction of 2-methoxybenzoyl chloride (0.42 g, 2.46 mmol) with freshly prepared sodium 2-methoxybenzene-carbotelluroate (0.749 g, 2.62 mmol) in acetonitrile (5 mL), followed by recrystallization from chloroform/petroleum ether gave 0.708 g (72%) of **4f** as yellow needles. M.p. 88 °C (dec.); IR (KBr) [cm^-1^]: 3,100, 3,000, 2,950, 2,850, 1,742 [C=O?], 1,675 [C=O], 1,625 [C=O], 1,590, 1,575, 1,480, 1,460, 1,445, 1,425, 1,305, 1,240, 1,175, 1,155, 1,100, 1,045, 1,010, 1,000, 840, 780, 745, 640, 590, 520, 490; ^1^H-NMR (270 MHz, CDCl_3_): δ = 3.95 (s, 3H) [C*H*_3_O], 6.98-7.62 (m, 8H) [arom]; ^13^C-NMR (67.8 MHz, CDCl_3_): δ = 55.4 [*C*H_3_O], 111.0, 121.0, 128.6, 132.0, 134.1, 158.6 [arom], 192.1 [*C*=O]; ^125^Te-NMR (85.9 MHz, CDCl_3_): δ = 1279.3; UV/Vis. (CH_2_Cl_2_) [nm]: 255, 313, 355 sh, 400 sh, 460; MS (CI, 70 eV): *m/z* = 367 [M^+^ +1], 395, 393. 

From potassium 2-methoxybenznecarbotelluroate*.* 82%. Yellow needles (recrystallization solvents: dichloromethane/petroleum ether). M.p. 88 °C (dec.). 

*Bis(4-methoxybenzoyl) telluride* (**4g**). 29%. Yellow needles (recrystallization solvents: dichloromethane/petroleum ether); m.p. 95–98°C (dec.); IR (KBr) [cm^-1^]: 3,026, 2,985, 2,925, 2,830, 1,716 [C=O], 1,702 [C=O], 1,595, 1,570. 1,505, 1,420, 1,290, 1,170, 1,155, 1,120, 1,095, 1,020, 915, 835, 810, 760, 675, 618, 598, 531, 490, 470; ^1^H-NMR (270 MHz, CDCl_3_): δ = 3.82 (s, 3H) [C*H*_3_], 6.87 (d, *J* = 9.8 Hz, 2H) [arom], 7.80 (d, *J* = 8.8 Hz, 2H) [arom]; ^13^C-NMR (67.8 MHz, CDCl_3_): δ = 55.7 [*C*H_3_], 114.4, 131.0, 132.3, 164.5 [arom], 181.6 [*C*=O]; ^125^Te-NMR (85.9 MHz, CDCl_3_): δ = 1070.9.

*Bis(2-chlorobenzoyl) telluride* (**4h**). M.p. 70 °C (dec.); Yellow needles (recrystallization solvents: dichloromethane/petroleum ether/ether). IR (KBr) [cm^-1^]: 3,010, 1,750 [C=O], 1,650 [C=O], 1,590, 1,465, 1,435, 1,250, 1,180, 1,100, 1,040, 960, 950, 865, 770, 745, 720, 685, 633, 590; ^1^H-NMR (270 MHz, CDCl_3_): δ = 7.17–7.31 (m, 8H) [arom]; ^13^C-NMR (67.8 MHz, CDCl_3_): δ = 126.8, 128.9, 130.6, 132.6, 140.9, 159.8 [arom], 192.1 [*C*=O]; ^125^Te-NMR (85.9 MHz, CDCl_3_): δ = 1294.7.

*Bis(4-chlorobenzoyl) telluride* (**4i**). 68%; Orange yellow micro-crystals; m.p. 75–78 °C ? (dec).; IR (KBr) [cm^-1^]: 3,028, 1,725 (C=O), 1,700 (C=O), 1,470, 1,120 m 1,040, 720; ^1^H-NMR (270 MHz, CDCl_3_): δ = 7.47 (d, *J* = 8.5 Hz, 2H) [arom], 7.74 (d, *J* = 8.5 Hz, 2H) [arom]; ^13^C-NMR (67.8 MHz, CDCl_3_): δ = 129.5, 129.6, 139.9, 141.2 [arom], 190.4 [*C*=O]; ^125^Te-NMR (85.9 MHz, CDCl_3_): δ = 1488.9.

*Bis(3-chloro-2,6-dimethoxybenzoyl) telluride* (**4j**). 39%; M.p. 89-91 °C (dec.) (recrystallization solvents: dichloromethane/petroleum ether); IR (KBr) [cm^-1^]: 3,025, 2,950, 2,850, 1,715 (C=O), 1,685 (C=O), 1,575, 1,560, 1,540, 1,465, 1,455, 1,430, 1,395, 1,365, 1,285, 1,270, 1,230, 1,170, 1,140, 1,080, 1,005, 1,000, 915, 895, 880, 800, 775, 725, 705, 680, 665, 640, 625, 575, 540, 490; ^1^H-NMR (270 MHz, CDCl_3_): δ = 3.81 (s, 6H) [C*H*_3_O], 3.86 (s, 6H) [C*H*_3_O], 6.62‒7.36 (m, 4H) [arom]; ^13^C-NMR (67.8 MHz, CDCl_3_): δ = 56.1 [*C*H_3_O], 62.6 [*C*H_3_O], 108.9, 119.9, 129.8, 132.5, 151.4, 154.8 [arom], 192.1 [*C*=O]; ^125^Te-NMR (85.9 MHz, CDCl_3_): δ = 1248.5; UV/Vis. (CH_2_Cl_2_) [nm]: 252, 285, 455.

*Di(1-naphthoyl) telluride* (**4k**). 84%. Orange yellow needles. M.p. 104 °C (dec.) (recrystallization solvents: dichloromethane/petroleum ether); IR (neat, CsI) [cm^-1^]: 3,075, 1,710 [C=O], 1,665 [C=O], 1,590, 1,575, 1,500, 1,210, 1,205, 1,195, 1,155, 1,040, 880, 870, 865, 800, 790, 765, 730, 715, 640, 625, 585, 555, 480; ^1^H-NMR (270 MHz, CDCl_3_): δ = 7.51–8.69 (m, 20H) [arom]; ^13^C-NMR (67.8 MHz, CDCl_3_): δ = 124.6, 125.1, 127.1, 127.4, 128.3, 128.9, 130.9, 132.1, 134.0, 139.5 [arom], 196.1 [*C*=O]; ^125^Te-NMR [85.9 MHz, CDCl3]: δ = 1176.4.

#### Reactions of compounds **4** with Grignard reagents

*With 4-methylphenylmagnesium bromide:* A solution of 4-methylphenylmagnesium bromide (0.396 N, 5 mL) in tetrahydrofuran was added to di(2-methoxybenzoyl) telluride (**4f**, 0.393 g, 1.0 mmol) in the same solvent (20 mL) at 0 °C and the mixture was stirred at the same temperature for 2 h. The orange yellow color [λ_max_ (CH_2_Cl_2_): 255, 313, 350 sh; λ_max_ (THF): 260, 315, 356 sh] quickly changed to dark green [UV-Vis (THF): λ_max_ 392 sh, 732 nm; ^13^C-NMR (67.8 MHz, THF-d_8_): δ = 232 [*C*=Te]. Water (30 mL) was added and stirred at 20 °C for 12 h until the green color changed to red orange [*Caution: very unpleasant smell (He_2_Te ?*)]. Dichloromethane (100 mL) was added and the organic layer washed with water (30 mL x 3), followed by drying with anhydrous sodium sulfate. Removal of the solvent under reduced pressure and preparative thin layer chromatography of the residue (eluent: dichloromethane/hexane = 3:1) gave 1,1-bis(4-methylphenyl)-1-(2-methoxyphenyl)-methanol (**11d**) (Rf = 0.08) as colorless micro-crystals. Yield: 0.235 g (84%); m.p. 123-125 °C; IR (KBr) [cm^-1^]: 3,450, 3,050, 2,975, 2,925, 2,850, 1,590, 1,575, 1,505, 1,480, 1,455, 1,425, 1,405, 1,350, 1,275, 1,235, 1,175, 1,150, 1,115, 1,015, 1,010, 915, 900, 815, 770, 750, 735, 650, 595, 575, 560, 495; ^1^H-NMR (270 MHz, CDCl_3_): δ = 2.32 (s, 6H) [C*H*_3_], 3.73 (s, 3H) [C*H*_3_O], 5.26 (s, 1H) [O*H*]; 6.6‒7.3 (m, 12H) [arom]; MS (EI, 20 eV) : *m/z* = 318 [M^+^], 301 [M –OH], 300 [M -H_2_O], 287 [M -CH_3_O]. 

*With 1-methylethylmagnesium bromide:* Compound **4f** (0,383 g, 1.0 mmol) and 1-methylethyl-magnesium bromide (0.57 N, 5 mL) were stirred in THF (15 mL) at 0 °C for 1 h. Water (20 mL) was added and stirred at 22 °C for 12 h. The reaction mixture was extracted with dichloromethane (2 x 50 mL), followed by drying over anhydrous sodium sulfate (ca. 5 g) for 1 h. The solvents were evaporated under reduced pressure. Thin layer chromatography (Rf = 0.16) of the resulting residue using a mixed solvent of dichloromethane/hexane (1:2) gave 1-(2-methoxyphenyl)-1-(1-methylethyl)methanol (**11a**) (Rf : 0.16); Yield: 0.072 g (24%) as a colorless oil; IR (KBr) [cm^-1^] 3,400 (br), 2,850, 2,925, 2,825, 1,485, 1,455, 1,430, 1,235, 1,020, 745; ^1^H-NMR (270 MHz, CDCl_3_): δ = 0.80 (d, 3H, *J* = 8.1 Hz) [C*H*_3_], 1.0 (d, 3H, *J* = 6.6 Hz) [C*H*_3_], 2.0 (sept, 1H, *J* = 10.1 Hz) [CH], 2.5 9 (br, 1H,) [O*H*], 3.80 (s, 3H) [C*H*_3_], 4.50 (d, 1H, *J* = 8.1 Hz) [C*H*], 6.83‒7.23 (m, 4H) [arom; MS (CI, 70 eV): *m/z* = 180 [M^+^], 162 [M-OH], 137 [2-MeOC6H4(OH)CH^+^], 107 [2-MeOC6H4 ^+^]. 

*With 1,1-dimethylethylmagnesium bromide:* The reaction of **4f** (0.800 g, 2.01 mmol) with 1,1-dimethylethylmagnesium bromide (0.603 N, 10 mL), followed by washing of the reaction mixture with water (50 mL) and evaporation of the THF under reduced pressure, gave bis(2-methoxybenzoyl) ditelluride (**12**) as red crystals. Yield: 0.363 g (69%); 128‒130 °C (dec); (lit. m.p. 130‒131°C, dec. [4b]); IR (KBr) [cm^-1^] 1620 (C=O); ^1^H-NMR (270 MHz, CDCl_3_): δ = 3.15 (s, 6H, C*H*_3_O). The IR spectrum of **12** was identical to that of an authentic sample prepared by the oxidation of piperidinium 2-methoxybenzenecarbotelluroate with iodine [4b].

*With 2-methoxyphenylmagnesium bromide:* Compound **4f** (0.402 g, 1.01 mmol) and 2-methoxy-phenylmagnesium bromide (0.304 N, 10 mL) were stirred in THF (20 mL) at 0 °C for 1 h, followed by treatment of the reaction mixture with water (50 mL) and by thin layer chromatographic separation as mentioned above to give 1,1,1-tris(2-methoxyphenyl)methanol (**11c**) as colorless crystals. Yield: 0.591 g (24%) as colorless crystals; m.p. 184‒185 °C; IR (KBr) [cm^-1^]: 3,560. 3,530, 2,975, 2,875, 2,850, 1,597, 1,580, 1,487, 1,465, 1,455, 1,430, 1,405, 1,285, 1,245, 1,230, 1,180, 1,160, 1,115, 1,105, 1,020, 1,000, 910, 900, 790, 755, 730, 625, 565; ^1^H-NMR (270 MHz, CDCl_3_): δ = 3.46 (s, 6H) [C*H*_3_], 3.73 (s, 3H) [C*H*_3_O], 5.26 (s, 1H) [O*H*], 6.6‒7.3 (m, 12H) [arom]; MS (CI, 70 eV) (*m/z* = 318 (M^+^), 301 (M -OH), 300 (M -H_2_O), 287 (M -CH_3_O).

## References

[1] Bergmann J., Engman L. (1978). Preparation of Selena- and Tellura Phthalic Anhydride. Org. Prep. Proc. Int..

[2] Severengiz T., du Mont W.W., Renoir D., Voss H. (1985). Novel Reactions of Acyl Halides with Bis(trimethylsilyl) Telluride: C, Te and C, C Bond Formation. Angew. Chem. Int. Ed. Engl..

[3] Sewing D., du Mont W.W., Pohl S., Saak W. (1998). Diacyltelluride: Synthesen durch Reaktionen von Acylchloriden mit Bis(trialkylsilyl)telluriden; Strukturbestimmunge an Di(1-adamantoyl)tellurid und Adamantancarbonsäureanhydrid. Z. Anorg. Allg. Chem..

[4] Kato S., Kakigano T., Ishida M. (1986). Tellurium Isologues of Acid Anhydrides: The first Acyclic Bis(acyl) Tellurides. Z. Chem..

[B5-molecules-14-02555] 5. The reactions appear to produce the telluride quantitatively and the low yield of **4d** is due to the loss during purification

[6] Bondi A. (1964). van der Waals Volumes and Radii. J. Phys. Chem..

[7] Minyaev R.S., Minkin V.I. (1998). Theoretical study of O→X (S, Se, Te) coordination in organic compounds. Can. J. Chem..

[B8-molecules-14-02555] 8. CCDC 719922 (**4f**), CCDC 719923 (**5**) and CCDC 719924 (**6**) contain the supplementary crystallographic data for this paper. These data can be obtained free of charge from The Cambridge Crystallographic Data Centre via www.ccdc.cam.ac.uk/data_request/cif.

[9] Kucsman A., Kapovits I. (1985). Organic Sulfur Chemistry: Theoretical and Experimental Advances.

[10] Niyomura O., Kato S., Inagaki S. (2000). An Unusual Planar Diacyl Ditelluride (2-MeOC_6_H_4_CO)_2_Te: The Origin of its Planarity. J. Am. Chem. Soc..

[11] Nishio M. (1998). The CH/π Interaction. Evidence, Nature, and Consequences.

[12] Huzinaga S., Andzelm J., Klobukowski M., Radzio-Andzelm Y., Sakai Y., Tatewaki H. (1984). Gaussian Basis Sets for Molecular Calculations.

[13] Frisch M.J., Trucks G.W., Schlegel H.B., Scuseria G.E., Robb M.A., Cheeseman J.R., Montgomery J.A. Jr., Vreven T., Kudin K.N., Burant J.C., Millam J.M., Iyengar S.S., Tomasi J., Barone V., Mennucci B., Cossi M., Scalmani G., Rega N., Petersson G.A., Nakatsuji H., Hada M., Ehara M., Toyota K., Fukuda R., Hasegawa J., Ishida M., Nakajima T., Honda Y., Kitao O., Nakai H., Klene M., Li X., Knox J.E., Hratchian H.P., Cross J.B., Adamo C., Jaramillo J., Gomperts R., Stratmann R.E., Yazyev O., Austin A.J., Cammi R., Pomelli C., Ochterski J.W., Ayala P.Y., Morokuma K., Voth G.A., Salvador P., Dannenberg J.J., Zakrzewski V.G., Dapprich S., Daniels A.D., Strain M.C., Farkas O., Malick D.K., Rabuck A.D., Raghavachari K., Foresman J.B., Ortiz J.V., Cui Q., Baboul A.G., Clifford S., Cioslowski J., Stefanov B.B., Liu G., Liashenko A., Piskorz P., Komaromi I., Martin R.L., Fox D.J., Keith T., Al-Laham M.A., Peng C.Y., Nanayakkara A., Challacombe M., Gill P.M.W., Johnson B., Chen W., Wong M.W., Gonzalez C., Pople J.A. (2003). Gaussian 2003.

[14] Kulkarni Y.D., Srivastava S., Athar M. (1986). Synthesis, Characterization & Antibacterial Actvity of Dibenzoyl Telluride & Heterocyclic Systems Derived from Them. Indian J. Chem..

[15] Becke A.D. (1993). Density-functional thermochemistry. III. The role of exact exchange. J. Chem. Phys..

[16] Lee C., Yang W., Parr R.G. (1988). Development of the Colle-Salvetti correlation-energy formula into a functional of the elrectron density. Phys. Rev. B.

[17] Duning T.H., Hay P.J., Schaefer H.F. (1977). Methods of Electronic Structure Theory.

[18] Acrey S.F. (1904). Ber. Deutsch. Chem. Ges..

[19] Kato T. (1972). Bachelor Thesis of Gifu University.

[20] Kawahara Y., Kato S., Kanda T., Murai T., Ebihara M. (1995). Synthesis of Rubidium and Cesium Tellurocarboxyaltes and an X-Ray Structural Analysis of Heavy Alkali Metal Monochalcogeno carbxoylates. Bull. Chem. Soc. Jpn..

[21] North A.C.T., Philips D.C., Mathews F.S. (1968). A semi-Empirical Method of Absorption Correction. Acta Crystallogr. Sect. A.

[22] Sheldrick G.M. (1997). SHELXL 97 Program for the Refinement of Crystal Structure.

[23] Cromer D.T., Waber J.T. (1974). International Tables for X-ray Crystallography.

[24] Creagh D.C., McAuley W. J., Wilson A.J.C. (1992). International Tables for X-ray Crystallography.

[25] Masumoto H., Tsutsumi H., Kanda T., Komada M., Murai T., Kato S. (1989). A Convenient Synthesis of Diacyl Sulfides. Sulfur Lett..

[26] Rambacher P., Mäke S. (1968). Verfahren zur Herstellung von Anhydriden aromatischer Carbonsäuren. Angew. Chem..

